# Tracking development assistance for health to fragile states: 2005–2011

**DOI:** 10.1186/s12992-015-0097-9

**Published:** 2015-03-19

**Authors:** Casey M Graves, Annie Haakenstad, Joseph L Dieleman

**Affiliations:** Institute for Health Metrics and Evaluation, University of Washington, 2301 5th Avenue, Suite 600, Seattle, WA 98121 USA

**Keywords:** Development assistance for health, Fragile states, Official development assistance, Disability-adjusted life years

## Abstract

**Background:**

Development assistance for health (DAH) has grown substantially, totaling more than $31.3 billion in 2013. However, the degree that countries with high concentrations of armed conflict, ethnic violence, inequality, debt, and corruption have received this health aid and how that assistance might be different from the funding provided to other countries has not been assessed.

**Methods:**

We combine DAH estimates and a multidimensional fragile states index for 2005 through 2011. We disaggregate and compare total DAH disbursed for fragile states versus stable states.

**Results:**

Between 2005 and 2011, DAH per person in fragile countries increased at an annualized rate of 5.4%. In 2011 DAH to fragile countries totaled $6.2 billion, which is $5.05 per person. This is 43% of total DAH that is traced to a country. Comparing low-income countries, funding channeled to fragile countries was $7.22 per person while stable countries received $11.15 per person. Relative to stable countries, donors preferred to provide more funding to low-income fragile countries that have refugees or ongoing external intervention but tended to avoid providing funding to countries with political gridlock, flawed elections, or economic decline. In 2011, Ethiopia received the most health aid of all fragile countries, while the United States provided the most funds to fragile countries.

**Conclusions:**

In 2011, 1.2 billion people lived in fragile countries. DAH can bolster health systems and might be especially valuable in providing long-term stability in fragile environments. While external health funding to these countries has increased since 2005, it is, in per person terms, almost half as much as the DAH provided to stable countries of comparable income levels.

**Electronic supplementary material:**

The online version of this article (doi:10.1186/s12992-015-0097-9) contains supplementary material, which is available to authorized users.

## Background

Development assistance for health (DAH) is an important contribution to health systems in many low- and middle-income countries. Over the last two decades, there has been immense growth in DAH, coinciding with the adoption of the Millennium Development Goals, as well as the onset of the HIV/AIDS epidemic and other global health challenges. Reaching an all-time high of $31.3 billion^a^ in 2013, DAH is now more than five times greater than it was in 1990 [1,2].

DAH is an especially important financing stream for health systems in fragile countries [3]. In many cases, these states lack sufficient health infrastructure, oversight and management, referral systems, and the ability to provide services outside of a few urban locations [3]. Furthermore, fragile states are characterized by outbreaks of epidemics, increased susceptibility to diseases, malnutrition, and increased barriers to access to health care [4]. In these settings, external resources are most often channeled to non-governmental organizations (NGOs) and fund primary health services [5].

Tracking development assistance to fragile countries is important to understanding whether health and health system support is provided despite the challenge of working in these areas. This research focuses exclusively on development assistance for health, rather than humanitarian aid, in an effort to compare funding streams that sustain the health sector of fragile and stable countries. While humanitarian aid is essential to maintaining the health of populations after natural disasters or during civil unrest, DAH can play a distinct and crucial role in supporting the long-term viability of the health sector of fragile states. A consistent stream of development aid can contribute to strengthening and maintaining a functioning health system and sustaining existing health services [6-9]. By focusing on DAH rather than humanitarian aid, we track donors’ long-term commitments to health in fragile states.

There are a number of studies that track development assistance and humanitarian relief to these states, although they generally assess aid at a single point in time, track total aid (rather than funding specifically for the health sector), include both humanitarian and development aid, assess aid to a single health focus area, or examine aid from a single funder [10-12]. This analysis complements these important studies by providing a comprehensive estimate of development assistance for the health sector, net of humanitarian aid, over time. We estimate the development assistance for the health sector transferred to fragile states from 2005 to 2011 and compare these funding flows with disbursements to stable countries.

## Methods

DAH to low- and middle-income countries is estimated and reported in the Institute for Health Metrics and Evaluation’s (IHME) *Financing Global Health 2013*: *Transition in an Age of Austerity* [1,2,13,14]. DAH is different from the official development assistance (ODA) reported by the Organisation for Economic Co-operation and Development (OECD) as it focuses exclusively on assistance targeting the health sector and tracks resources from a broader set of donors, including bilateral and multilateral aid organizations, NGOs, private foundations based in the US, and other entities [15]. IHME defines DAH as all financial and in-kind contributions primarily intended for the health sector. This breadth, combined with the unique health-sector focus, makes these data particularly suitable for assessing the health aid disbursed to fragile states for non-emergency humanitarian purposes.

The main data sources used to produce the *Financing Global Health 2013* DAH database include revenue and expenditure data from the OECD Creditor Reporting System (CRS), channel-specific project databases, annual reports, financial statements, tax filings, budget documents, and personal correspondence. In the simplest cases, DAH is transferred from a funding source to an intermediary channel and from an intermediary channel to an implementing agency. However, funds are often transferred between channels. IHME corrects for transfers between channels to avoid double-counting. When disbursement data are not available, various estimation methods based on commitments or appropriations data are used. This research made use of existing data, and thus was not human subjects relevant.

There has been little consensus on how to define fragile states, despite a working definition proposed by the United Kingdom’s Department for International Development (DFID) as early as 2005 [16]. Over 10 publicly available fragility indices are produced by numerous development agencies, research institutes, and academic groups [17]. We utilized the Fragile States Index from the Fund for Peace (FFP) to distinguish between fragile and stable countries [18]. FFP uses expert opinion and quantitative and qualitative evidence to generate annual scores across 12 social, economic, political, and military indicators for a large set of countries and has been subject to numerous peer reviews since it was first developed in the 1990s [19]. Each indicator is assigned a score from zero to 10 to signify the level of various pressures within each country, along with an aggregated score across the 12 indicators. FFP does not delineate fragile and stable states, but rather considers those with an aggregate score greater than 90 to have “alert” status, defined as vulnerable to conflict or collapse. States within this category are characterized by a loss of control of territory, erosion of legitimate authority, and the inability to provide public services and interact with the international community [20]. We followed FFP’s conventional threshold and label a fragile country as any nation that received an aggregate score greater than 90 or an individual indicator score greater than 7.5. All other countries were considered stable. Rigor, multidimensionality, and 10 years of estimates make the FFP index uniquely appropriate for this analysis.

FFP’s fragility data is available from 2005 to 2014, while IHME’s country-level DAH estimates are available from 1990 to 2011. Thus, our combined dataset ranges from 2005 to 2011. 141 countries are assessed in both the fragility and DAH datasets, and are thus included in this analysis. When pertinent, we stratified countries into income groups based on contemporaneous World Bank income-group classifications. The FFP index is also employed in a contemporaneous manner, such that the categories reflect the countries with those characteristics for a given year, and thus the countries do change from year to year, based on their categorization. IHME does not report DAH to high-income countries, which results in the removal of 11 countries from the database for the years in which they were considered high-income. Additional file 1: Table S1 provides the list of country classifications from 2005 to 2011. Across the seven-year time period, 28 to 37 countries were classified as fragile.

## Results

In 2011, $6.2 billion of development assistance for health was allocated to fragile countries. This is 43% of the DAH that can be traced to a single country. 1.2 billion people lived in fragile countries in 2011, putting DAH levels of $5.05 per person. While the population of people living in fragile countries has increased since 2005, the amount of DAH going to these countries has increased faster. In 2005, DAH disbursed in fragile countries amounted to $3.69 per person. In contrast, stable countries received $1.79 per person in 2011. This relationship is primarily because less DAH per person is transferred to middle-income countries, while the majority of fragile countries are low-income. When we compare only low-income countries, we see that per person disbursements to fragile countries are in fact less than transfers to stable countries. Low-income fragile countries received $7.22 per person, while low-income stable countries received $11.15 per person in 2011. This distinct difference in per person disbursement levels has existed since 2007, with stable countries receiving an average of $5.05 more per person than fragile countries. Before 2007, low-income fragile countries received more per capita than low-income stable countries.

**Figure 1:**
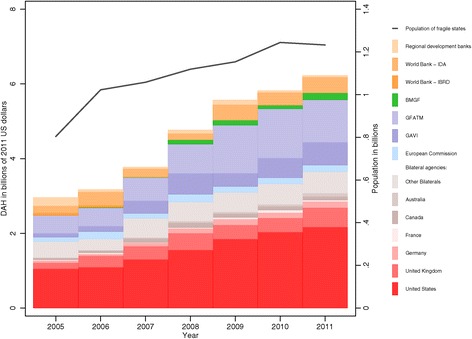
**Total population in fragile countries and development assistance for health to fragile countries by channel**, **2005–2011.** Development assistance for health is reported in 2011 US dollars. Population is total population in fragile states. Regional development banks include the Asian Development Bank, African Development Bank, and Inter-American Development Bank. Other bilaterals include Austria, Belgium, Denmark, Finland, Greece, Ireland, Italy, Japan, South Korea, Luxembourg, the Netherlands, Norway, New Zealand, Portugal, Spain, Sweden, and Switzerland. IBRD = International Bank for Reconstruction and Development. IDA = International Development Association. BMGF = Bill & Melinda Gates Foundation. GFATM = Global Fund to Fight AIDS, Tuberculosis and Malaria. GAVI = Gavi, The Vaccine Alliance.

Figures 1 shows the agencies, organizations, and foundations that channeled DAH to all fragile countries from 2005 to 2011. Over the time period, total DAH to fragile countries increased at an annualized rate of 13.2%, while total population in these countries grew only 7.4% annually. The United States disbursed more DAH than any other channel. In 2011, the US channeled $2.2 billion, or 35% of total DAH disbursed to fragile countries. The Global Fund to Fight AIDS, Tuberculosis and Malaria (GFATM) also consistently provided a significant share of the DAH for fragile countries, contributing 18% of DAH to fragile countries in 2011.

**Figure 2:**
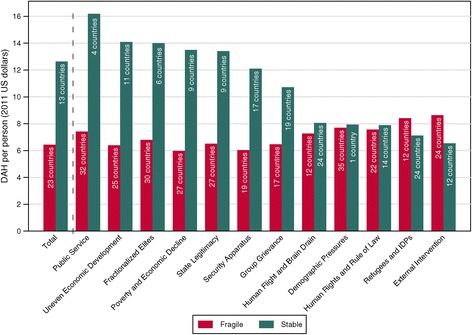
**Development assistance for health per person to low**-**income countries by Fragile States Index score**, **2009**–**2011**. Total development assistance for health (DAH) per person is averaged across 2009–2011. Fragile is defined as having a Fund for Peace total score greater than 90 or a score greater than 7.5 for one of the 12 indicators. The bar labels indicate how many countries are included in each category. Countries are included if they were low-income for at least two of the three years.

Figure 2 disaggregates DAH per person to fragile low-income countries by each of the 12 indicators making up FFP’s Fragile Countries Index. These indicators make up the components of the composite Fragile Countries Index. Figure 2 shows each country’s designation per each of these indicators (some countries may be classified as fragile by a given indicator, but are categorized as stable overall). Poor public services, uneven economic development, fractionalized elites, and poverty and economic decline had the strongest relationship with DAH per person, as countries with these characteristics received between $8.76 and $7.21 less per person than countries without these characteristics. Demographic pressures, human rights and rule of law, and human flight and brain drain, on the other hand, had a weak relationship with the amount of DAH per person received. Lastly, external intervention and refugees and internally displaced persons (IDPs) seemed to attract support for health, as countries with these issues received up to $2.09 more per person than the other countries.

**Figure 3:**
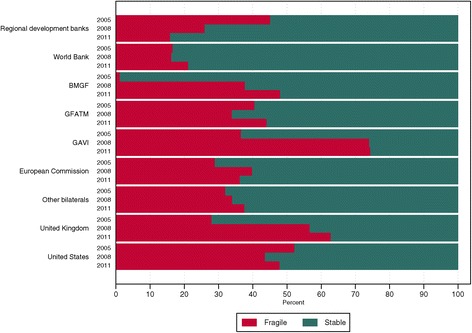
**Development assistance for health composition by channel**, **2005**, **2008**, **and 2011.** Development assistance for health allocable to fragile (defined as having a Fund for Peace score above 90) and stable (defined as having a Fund for Peace score less than or equal to 90) countries. Regional development banks include the Asian Development Bank, African Development Bank, and Inter-American Development Bank. World Bank includes International Bank for Reconstruction and Development and International Development Association. Other bilaterals include Austria, Belgium, Denmark, Finland, Greece, Ireland, Italy, Japan, South Korea, Luxembourg, the Netherlands, Norway, New Zealand, Portugal, Spain, Sweden, and Switzerland. Global Fund = Global Fund to Fight AIDS, Tuberculosis and Malaria. GAVI = Gavi, the Vaccine Alliance.

Figure 3 characterizes donors’ disbursement patterns and illustrates the share of each channel’s total DAH to fragile countries and stable countries in 2005, 2008, and 2011. Among the DAH we can allocate to fragile and stable countries, the Gavi, the Vaccine Alliance provided the largest share of its funds to fragile countries, contributing 74% in 2011. The United Kingdom and the United States also provided a substantial portion of their funds to these countries in 2011, allocating 62% and 48% of their respective DAH disbursements to fragile countries.

A number of large sub-Saharan African countries classified as fragile received substantial volumes of DAH, while a few small island countries received high levels of DAH per capita. In 2011 alone, Ethiopia received the most health aid. However, across the entire period, Nigeria received more DAH than any other fragile state (even excluding DAH received in 2005, when Nigeria was not considered fragile by FFP). On the other hand, in per capita terms, Solomon Islands and Timor-Leste received more than four times as much DAH as Nigeria. These countries received, on average, $28.81 and $19.58 per capita, respectively. The fact that countries with small populations receive disproportionally large quantities of DAH per capita has been noted elsewhere as well [21,22]. In fragile countries, DAH targeted for HIV/AIDS, maternal, newborn, and child health, and malaria was 38%, 25%, and 11% of the total, respectively. These disbursement patterns are similar to disbursement patterns of stable countries.

**Figure 4:**
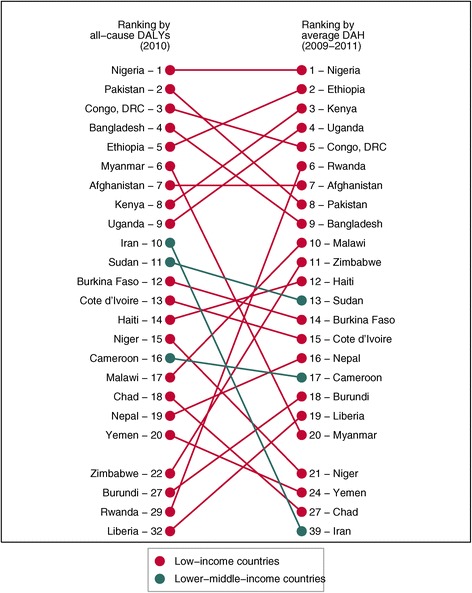
**Burden of disease compared to development assistance for health for fragile countries**, **2009**–**2011.** Burden of disease, defined as all-cause disability-adjusted life years (DALYs) (2010), and average development assistance for health from 2009–2011 for all fragile states (defined as having a Fund for Peace score greater than 90 or an individual indicator score greater than 7.5). Countries are color coded by World Bank income classification.

Finally, Figure 4 provides two lists of fragile countries, the first showing the top 20 DAH recipients and the second showing the largest disease burdens, measured by all-cause disability-adjusted life years (DALYs) [23]. The figure shows that DAH and disease burden are positively associated for most fragile countries. Countries such as Nigeria and Ethiopia have very large disease burdens and received more DAH than other fragile countries. However, disparity exists for several fragile countries, including Myanmar, Iran, and Chad. These countries received less DAH than their disease burden would suggest.

## Discussion

A substantial and growing amount of DAH was provided to countries classified as fragile from 2005 to 2011. When examining disbursements to low- and middle-income stable countries, fragile countries received more in per capita terms. However, when we focus on the low-income group only, fragile countries received less in per capita terms. In 2011, a gap of $3.93 per person separates DAH received by fragile and the DAH received by stable low-income countries. This gap underscores that less global health aid is provided to countries under political and economic stress.

Further investigation reveals that while, on average, low-income fragile countries receive less DAH per person than comparable stable countries, donors prioritize countries with refugees, displaced persons, and existing external engagement. On the other hand, donors have provided less long-term development assistance for health to countries with poor public services, uneven economic development, fractionalized elites, and economic decline, as these countries receive disproportionally less DAH.

While operating in fragile countries is both risky and costly, our data highlight donors’ increasing provision of funds. External funding can be an important input to bolstering health systems and delivering health care in unstable political environments. This research shows that development assistance partners are not veering away from providing funds to fragile countries; while they provide less, vis-à-vis stable countries, they have, in fact, increased their investments in fragile countries over time. This may indicate a lag between the point at which a country becomes fragile and the disbursement of DAH, indicating that donors may, in fact, be responding as states fall into fragility.

While this analysis provides a unique perspective on trends in DAH to fragile and stable countries, data limitations prevent us from subnational analyses, and thus we are unable to pinpoint precisely where funds are being disbursed. Consequently, we cannot determine if the resources are destined for fragile sub-regions within a country. Additionally, IHME is not able to allocate all DAH to a recipient country since several channels do not report recipient-level data or report funds as going to a region or as having a global focus. Due to lags in reporting, our DAH by recipient data only extend through 2011.

## Conclusions

Our analysis highlights that while development assistance partners have increased funding to low-income fragile countries since 2005, they have not provided funds to these populations at the same rate as in stable low-income countries. More analysis is required, but this may point to the fact that development assistance is provided more in the form of humanitarian assistance than as DAH in times of stress. Still, DAH is a unique and important means to achieving long-term sustainability in the health sector. Future analysis should focus on improving the understanding of the role of development assistance partners in health during times of crisis, which is important to ensuring health systems do not fail when countries face epidemics or other health challenges, as well as internal strife and political and economic crises.

### Endnotes

^a^All currency estimates reported in real, 2011 US dollars.

## Additional file

Additional file 1: Table S1. Fragile States Index classifications of 141 countries, 2005–2011. Detailed list of Fragile States Index classifications for all countries in the analysis from 2005 to 2011. 0 = stable, 1 = fragile. HIC indicates the country is classified as high income. A dash indicates the country was not included in the Fragile States Index and is, by default, considered stable.

## Abbreviations

CRS: Creditor Reporting System
DAH: Development assistance for health
DALY: Disability adjusted life year
DFID: United Kingdom’s Department for International Development
FFP: Fund for Peace
GDP: Gross domestic product
GFATM: Global Fund to Fight AIDS, Tuberculosis and Malaria
IHME: Institute for Health Metrics and Evaluations
NGO: Non-governmental organizations
OECD: Organisation for Economic Cooperation and Development
