# Supplementary material for: Tracking development assistance for health to fragile states: 2005–2011
Source: Global Health. 2015 Mar 19;11:12. doi: 10.1186/s12992-015-0097-9 (PMC4381367; doi:10.1186/s12992-015-0097-9)
Supplement: Additional file 1: Table S1. — Fragile States Index classifications of 141 countries, 2005–2011. Detailed list of Fragile States Index classifications for all countries in the analysis from 2005 to 2011. 0 = stable, 1 = fragile. HIC indicates the country is classified as high income. A dash indicates the country was not included in the Fragile States Index and is, by default, considered stable. [file 12992_2015_97_MOESM1_ESM.pdf]

Tracking development assistance for health to fragile states: 2005-2011
Authors: Casey Graves, Annie Haakenstad, Joseph L Dieleman

**Additional file 1. Table S1: Fragile States Index classifications of 141 countries, 2005-2011**
0 = stable, 1 = fragile. HIC indicates the country is classified as high income.
A dash indicates the country was not included in the Fragile States Index and is, by default, considered stable.

| Country | 2005 | 2006 | 2007 | 2008 | 2009 | 2010 | 2011 |
|---|---|---|---|---|---|---|---|
| Afghanistan | 1 | 1 | 1 | 1 | 1 | 1 | 1 |
| Albania | - | 0 | 0 | 0 | 0 | 0 | 0 |
| Algeria | 0 | 0 | 0 | 0 | 0 | 0 | 0 |
| Angola | 0 | 0 | 0 | 0 | 0 | 0 | 0 |
| Antigua & Barbuda | HIC | HIC | HIC | HIC | 0 | 0 | 0 |
| Argentina | - | 0 | 0 | 0 | 0 | 0 | 0 |
| Armenia | - | 0 | 0 | 0 | 0 | 0 | 0 |
| Azerbaijan | 0 | 0 | 0 | 0 | 0 | 0 | 0 |
| Bangladesh | 1 | 1 | 1 | 1 | 1 | 1 | 1 |
| Barbados | - | HIC | HIC | HIC | HIC | HIC | HIC |
| Belarus | 0 | 0 | 0 | 0 | 0 | 0 | 0 |
| Belize | - | - | 0 | 0 | 0 | 0 | 0 |
| Benin | - | 0 | 0 | 0 | 0 | 0 | 0 |
| Bhutan | 1 | 0 | 0 | 0 | 0 | 0 | 0 |
| Bolivia | - | 0 | 0 | 0 | 0 | 0 | 0 |
| Bosnia & Herzegovina | 1 | 0 | 0 | 0 | 0 | 0 | 0 |
| Botswana | - | 0 | 0 | 0 | 0 | 0 | 0 |
| Brazil | 0 | 0 | 0 | 0 | 0 | 0 | 0 |
| Bulgaria | - | 0 | 0 | 0 | 0 | 0 | 0 |
| Burkina Faso | - | 0 | 0 | 0 | 1 | 1 | 0 |
| Burundi | 1 | 1 | 1 | 1 | 1 | 1 | 1 |
| Cambodia | - | 0 | 0 | 0 | 0 | 0 | 0 |
| Cameroon | 0 | 0 | 0 | 1 | 1 | 1 | 1 |
| Cape Verde | - | - | 0 | 0 | 0 | 0 | 0 |
| Central African Republic | 1 | 1 | 1 | 1 | 1 | 1 | 1 |
| Chad | 1 | 1 | 1 | 1 | 1 | 1 | 1 |
| Chile | - | 0 | 0 | 0 | 0 | 0 | 0 |
| China | 0 | 0 | 0 | 0 | 0 | 0 | 0 |
| Colombia | 1 | 1 | 0 | 0 | 0 | 0 | 0 |
| Comoros | - | - | 0 | 0 | 0 | 0 | 0 |
| Congo | - | - | 1 | 1 | 1 | 1 | 1 |
| Congo, DRC | 1 | 1 | 1 | 1 | 1 | 1 | 1 |
| Costa Rica | - | 0 | 0 | 0 | 0 | 0 | 0 |
| Cote d'Ivoire | 1 | 1 | 1 | 1 | 1 | 1 | 1 |
| Croatia | - | 0 | 0 | HIC | HIC | HIC | HIC |
| Cuba | 0 | 0 | 0 | 0 | 0 | 0 | 0 |
| Djibouti | - | - | 0 | 0 | 0 | 0 | 0 |
| Dominican Republic | 1 | 0 | 0 | 0 | 0 | 0 | 0 |
| Ecuador | 0 | 0 | 0 | 0 | 0 | 0 | 0 |
| Egypt | 0 | 0 | 0 | 0 | 0 | 0 | 0 |
| El Salvador | 0 | 0 | 0 | 0 | 0 | 0 | 0 |
| Equatorial Guinea | 1 | 0 | HIC | HIC | HIC | HIC | HIC |
| Eritrea | 0 | 0 | 0 | 0 | 1 | 1 | 1 |
| Estonia | - | HIC | HIC | HIC | HIC | HIC | HIC |
| Ethiopia | 1 | 1 | 1 | 1 | 1 | 1 | 1 |
| Fiji | - | - | 0 | 0 | 0 | 0 | 0 |
| Gabon | - | 0 | 0 | 0 | 0 | 0 | 0 |
| Georgia | - | 0 | 0 | 0 | 1 | 1 | 0 |
| Ghana | - | 0 | 0 | 0 | 0 | 0 | 0 |
| Grenada | - | - | 0 | 0 | 0 | 0 | 0 |
| Guatemala | 1 | 0 | 0 | 0 | 0 | 0 | 0 |
| Guinea | 1 | 1 | 1 | 1 | 1 | 1 | 1 |
| Guinea-Bissau | - | 0 | 0 | 1 | 1 | 1 | 1 |
| Guyana | - | - | 0 | 0 | 0 | 0 | 0 |
| Haiti | 1 | 1 | 1 | 1 | 1 | 1 | 1 |
| Honduras | 0 | 0 | 0 | 0 | 0 | 0 | 0 |

| | | | | | | | |
|---|---|---|---|---|---|---|---|
| Hungary | - | 0 | HIC | HIC | HIC | HIC | HIC |
| India | 0 | 0 | 0 | 0 | 0 | 0 | 0 |
| Indonesia | 0 | 0 | 0 | 0 | 0 | 0 | 0 |
| Iran | 0 | 0 | 0 | 0 | 0 | 1 | 1 |
| Iraq | 1 | 1 | 1 | 1 | 1 | 1 | 1 |
| Jamaica | - | 0 | 0 | 0 | 0 | 0 | 0 |
| Jordan | - | 0 | 0 | 0 | 0 | 0 | 0 |
| Kazakhstan | 0 | 0 | 0 | 0 | 0 | 0 | 0 |
| Kenya | 1 | 0 | 1 | 1 | 1 | 1 | 1 |
| Kyrgyzstan | 0 | 1 | 0 | 0 | 0 | 0 | 1 |
| Laos | 1 | 0 | 0 | 0 | 0 | 0 | 0 |
| Latvia | - | 0 | 0 | 0 | HIC | 0 | 0 |
| Lebanon | 0 | 0 | 1 | 1 | 1 | 1 | 0 |
| Lesotho | - | - | 0 | 0 | 0 | 0 | 0 |
| Liberia | 1 | 1 | 1 | 1 | 1 | 1 | 1 |
| Libya | 0 | 0 | 0 | 0 | 0 | 0 | 0 |
| Lithuania | - | 0 | 0 | 0 | 0 | 0 | 0 |
| Macedonia | - | 0 | 0 | 0 | 0 | 0 | 0 |
| Madagascar | - | - | 0 | 0 | 0 | 0 | 0 |
| Malawi | - | 0 | 1 | 1 | 1 | 1 | 1 |
| Malaysia | 0 | 0 | 0 | 0 | 0 | 0 | 0 |
| Maldives | - | - | 0 | 0 | 0 | 0 | 0 |
| Mali | - | 0 | 0 | 0 | 0 | 0 | 0 |
| Mauritania | - | 0 | 0 | 0 | 0 | 0 | 0 |
| Mauritius | - | 0 | 0 | 0 | 0 | 0 | 0 |
| Mexico | 0 | 0 | 0 | 0 | 0 | 0 | 0 |
| Micronesia | - | - | 0 | 0 | 0 | 0 | 0 |
| Moldova | - | 0 | 0 | 0 | 0 | 0 | 0 |
| Mongolia | - | 0 | 0 | 0 | 0 | 0 | 0 |
| Montenegro | - | - | 0 | 0 | 0 | 0 | 0 |
| Morocco | 0 | 0 | 0 | 0 | 0 | 0 | 0 |
| Mozambique | 0 | 0 | 0 | 0 | 0 | 0 | 0 |
| Myanmar | 1 | 1 | 1 | 1 | 1 | 1 | 1 |
| Namibia | - | 0 | 0 | 0 | 0 | 0 | 0 |
| Nepal | 0 | 1 | 1 | 1 | 1 | 1 | 1 |
| Nicaragua | - | 0 | 0 | 0 | 0 | 0 | 0 |
| Niger | - | 0 | 1 | 1 | 1 | 1 | 1 |
| Nigeria | 0 | 1 | 1 | 1 | 1 | 1 | 1 |
| North Korea | 1 | 1 | 1 | 1 | 1 | 1 | 1 |
| Oman | - | 0 | HIC | HIC | HIC | HIC | HIC |
| Pakistan | 0 | 1 | 1 | 1 | 1 | 1 | 1 |
| Palestine | - | - | - | - | 0 | 0 | 0 |
| Panama | - | 0 | 0 | 0 | 0 | 0 | 0 |
| Papua New Guinea | - | 0 | 0 | 0 | 0 | 0 | 0 |
| Paraguay | 0 | 0 | 0 | 0 | 0 | 0 | 0 |
| Peru | 0 | 0 | 0 | 0 | 0 | 0 | 0 |
| Philippines | 0 | 0 | 0 | 0 | 0 | 0 | 0 |
| Poland | - | 0 | 0 | 0 | HIC | HIC | HIC |
| Romania | - | 0 | 0 | 0 | 0 | 0 | 0 |
| Russia | 0 | 0 | 0 | 0 | 0 | 0 | 0 |
| Rwanda | 1 | 1 | 0 | 0 | 0 | 0 | 1 |
| Samoa | - | - | 0 | 0 | 0 | 0 | 0 |
| Sao Tome & Principe | - | - | 0 | 0 | 0 | 0 | 0 |
| Senegal | - | 0 | 0 | 0 | 0 | 0 | 0 |
| Serbia | - | - | 0 | 0 | 0 | 0 | 0 |
| Seychelles | - | - | 0 | 0 | 0 | 0 | 0 |
| Sierra Leone | 1 | 1 | 1 | 1 | 1 | 1 | 1 |
| Slovakia | - | 0 | HIC | HIC | HIC | HIC | HIC |
| Solomon Is. | - | - | 1 | 1 | 0 | 0 | 0 |
| Somalia | 1 | 1 | 1 | 1 | 1 | 1 | 1 |
| South Africa | - | 0 | 0 | 0 | 0 | 0 | 0 |
| Sri Lanka | 0 | 1 | 1 | 1 | 1 | 1 | 1 |
| Sudan | 1 | 1 | 1 | 1 | 1 | 1 | 1 |
| Suriname | - | - | 0 | 0 | 0 | 0 | 0 |
| Swaziland | - | - | 0 | 0 | 0 | 0 | 0 |
| Syria | 1 | 0 | 0 | 1 | 0 | 0 | 0 |
| Tajikistan | 0 | 0 | 0 | 0 | 1 | 0 | 0 |

| | | | | | | | |
|---|---|---|---|---|---|---|---|
| Tanzania | 1 | 0 | 0 | 0 | 0 | 0 | 0 |
| Thailand | 0 | 0 | 0 | 0 | 0 | 0 | 0 |
| The Gambia | 0 | 0 | 0 | 0 | 0 | 0 | 0 |
| Timor Leste | - | - | 1 | 1 | 1 | 1 | 1 |
| Togo | 0 | 0 | 0 | 0 | 0 | 0 | 0 |
| Trinidad & Tobago | - | HIC | HIC | HIC | HIC | HIC | HIC |
| Tunisia | 0 | 0 | 0 | 0 | 0 | 0 | 0 |
| Turkey | 0 | 0 | 0 | 0 | 0 | 0 | 0 |
| Turkmenistan | 0 | 0 | 0 | 0 | 0 | 0 | 0 |
| Uganda | 1 | 1 | 1 | 1 | 1 | 1 | 1 |
| Ukraine | 0 | 0 | 0 | 0 | 0 | 0 | 0 |
| Uruguay | - | 0 | 0 | 0 | 0 | 0 | 0 |
| Uzbekistan | 1 | 1 | 1 | 1 | 1 | 1 | 0 |
| Venezuela | 1 | 0 | 0 | 0 | 0 | 0 | 0 |
| Vietnam | 0 | 0 | 0 | 0 | 0 | 0 | 0 |
| Yemen | 1 | 1 | 1 | 1 | 1 | 1 | 1 |
| Zambia | - | 0 | 0 | 0 | 0 | 0 | 0 |
| Zimbabwe | 1 | 1 | 1 | 1 | 1 | 1 | 1 |
| **Total fragile states** | **33** | **28** | **32** | **35** | **37** | **37** | **35** |
